# Serum Zinc Level Grading System: A Useful Model for Composite Hepatic Events in Hepatitis C Virus-Associated Liver Cirrhosis

**DOI:** 10.3390/jcm9030643

**Published:** 2020-02-28

**Authors:** Hiroki Nishikawa, Hirayuki Enomoto, Kazunori Yoh, Yoshinori Iwata, Yoshiyuki Sakai, Kyohei Kishino, Yoshihiro Shimono, Naoto Ikeda, Tomoyuki Takashima, Nobuhiro Aizawa, Ryo Takata, Kunihiro Hasegawa, Noriko Ishii, Yukihisa Yuri, Takashi Nishimura, Hiroko Iijima, Shuhei Nishiguchi

**Affiliations:** 1 Division of Hepatobiliary and Pancreatic Disease, Department of Internal Medicine, Hyogo College of Medicine, Nishinomiya, Hyogo 663-8501, Japan; 2 Center for Clinical Research and Education, Hyogo College of Medicine, Nishinomiya, Hyogo 663-8501, Japan

**Keywords:** serum zinc, classification system, liver cirrhosis, Child–Pugh classification, ALBI grade, composite hepatic events

## Abstract

We aimed to clarify the impact of the serum zinc (Zn) level grading system proposed by the Japanese society of clinical nutrition (JSCN: 80 μg/dL ≤ serum Zn level < 130 μg/dL (type A), 60 μg/dL ≤ serum Zn level < 80 μg/dL (type B), and serum Zn level < 60 μg/dL (type C)) in patients with hepatitis C virus (HCV)-related liver cirrhosis (LC) on the incidence of composite hepatic events (Com-HEs) compared with Child–Pugh (C–P) classification or albumin-bilirubin (ALBI) grade. (n = 275, median age = 67 years). The Akaike information criterion (AIC) was compared among three prognostic models. Factors associated with the incidence of Com-HEs were also studied. The first incidence of any HE was confirmed in 112 patients (40.7%). The AIC value for Com-HEs by the Zn level grading system was the lowest among the three prognostic models (AIC: 301.788 in Zn level grading system, 303.372 in ALBI grade, and 333.953 in C–P classification). In the multivariate analysis, male (*p* = 0.0031), ALBI grade 3 (*p* = 0.0041), type B (*p* = 0.0238), type C (*p* = 0.0004), and persistent viremia (*p* < 0.0001) were significant factors associated with the incidence of Com-HEs. In conclusion, the serum Zn level grading system proposed by JSCN can be helpful for estimating the incidence of Com-HEs in HCV-related LC patients.

## 1. Introduction

Liver cirrhosis (LC) is often accompanied by many clinical manifestations, including liver failure, hepatic encephalopathy (HE), ascites with or without infection, varices caused by portal hypertension, or liver carcinogenesis, all of which can cause adverse clinical outcomes [1,2,3,4]. The Child–Pugh (C–P) classification system is a globally accepted evaluation system for the hepatic reserve in patients with LC [5]. However, a major problem of the C–P classification system is that it involves two subjective elements (ascites and encephalopathy) and interrelated elements (serum albumin and ascites) [5]. For the purpose of overcoming these obstacles, the albumin-bilirubin (ALBI) grade, calculated by only serum albumin levels and total bilirubin levels, was proposed and has been extensively validated in numerous studies [6,7,8,9,10,11,12,13,14]. Taking outcomes into consideration in patients with LC, composite hepatic events (Com-HEs) have been widely used in the clinical setting [15,16,17,18,19]. Composite outcomes are indicators that combine several outcomes into a single measurement [20]. At the stage of LC, patients are at elevated risk of developing HEs, which shortens their life expectancy [15,16,17,18,19]. Composite study endpoints are preferentially used mainly in the field of cardiovascular diseases [21,22,23].

In the human body, zinc (Zn) is widely distributed, and the richest Zn is observed in muscle (about 60%) [24]. Zn is an important trace element for the development of normal cells, proliferation, and differentiation. Zn in the human body has an important role as a cofactor for enzymatic reactions, regulation of inflammation, and wound healing [25,26,27]. Zn deficiency impairs phagocytic function, and decreases lymphocyte count, immunoglobulin production, and interleukin-2 production [25,26,27]. Zn deficiency can lead to numerous disorders, such as dermatitis, hair loss, anemia, stomatitis, sexual dysfunction in male, abnormal taste, susceptibility, and osteoporosis [25,26,27]. LC patients tend to have lower serum Zn levels, and decreased serum Zn levels can be linked to a poor quality of life in LC patients [28]. Possible causes for the Zn decline in LC patients include impaired Zn absorption from the digestive tract, decreased Zn intake, and increased Zn excretion in the urine [27]. Hypozincemia has been associated with hepatocellular carcinoma (HCC) occurrence in patients with hepatitis C virus (HCV)-related LC [29]. Oral Zn supplementation treatment can reduce the incidence of LC-related HEs [27,30].

Currently, the Japanese society of clinical nutrition (JSCN) defines serum Zn level <60 μg/dL as having Zn deficiency, 60 μg/dL ≤ serum Zn level < 80 μg/dL as having subclinical Zn deficiency, and 80 μg/dL ≤ serum Zn level < 130 μg/dL as having a normal Zn value. In our previous investigation, we reported that the serum Zn grading system proposed by JSCN can be more useful for predicting overall survival (OS) in patients with LC compared with the Child–Pugh classification system or ALBI grading system using the Akaike information criterion (AIC) [31]. However, the impact of this Zn level grading system in patients with HCV-related LC on the incidence of Com-HEs remains unclear. In order to clarify these essential research issues, we compared the prognostic accuracy for Com-HEs among the serum Zn grading system, ALBI grading system, and Child–Pugh classification system in patients with HCV-related LC.

## 2. Patients and Methods

### 2.1. Patients

Between April 2008 and December 2018, 310 HCV-related LC subjects for whom data for baseline serum Zn levels were available were admitted at our institution. LC was determined by pathological data, imaging findings, and/or laboratory data [32,33,34]. No patient had obvious evidence of hepatitis B virus coinfection or HIV coinfection. HCV genotype was tested as reported previously [35]. HCV RNA concentration was tested in the TaqMan HCV assay (COBAS TaqMan HCV assay; Roche Molecular Diagnostics, Tokyo, Japan). Five patients were lost to follow-up within one year and were excluded. In the remaining 305 patients, 30 patients with HCC on their radiologic findings at baseline were excluded. A total of 275 subjects were thus analyzed in this analysis. The same baseline patient data as our previous reports [31] were used for analysis in cases where data was available.

The ALBI score was calculated and graded (ALBI grade 1, 2, and 3) as reported elsewhere [6]. In the observation period, blood tests and radiological tests, with the aim of detecting HCC development or LC-related complications, were done every 3–6 months. When the serum albumin level was below 3.5 g/dL, branched-chain amino acid therapies were started [36,37]. Antiviral treatments (i.e., direct-acting antivirals (DAAs) or interferon (IFN)-based therapies) for underlying HCV infection were started when indicated. In cases with hypozincemia, Zn supplementation therapy was started. In terms of HCC diagnosis and HCC treatment strategies, we principally conformed to the current standard guidelines [38,39].

### 2.2. Primary Outcome Measure in This Study

The primary endpoint in this study was the first incidence of Com-HEs [15,16,17,18,19]. Com-HEs included incidence of hepatic decompensation, HCC, new onset or deterioration of ascites, variceal bleeding, HE, or acute on chronic liver failure (ACLF). The diagnosis of any CHE was determined based on the current guidelines, and in cases with CHE incidence, an appropriate therapy was performed considering the current guidelines [37,40]. During the observation period, any Com-HE was registered. Patients were followed from the measurement of baseline serum Zn levels until the first incidence of Com-HEs, death, or until the end of follow-up.

### 2.3. Serum Zn Grading System and Our Study

Subjects with 80 μg/dL ≤ serum Zn level < 130 μg/dL at baseline were defined as type A (normal serum Zn level), those with 60 μg/dL ≤ serum Zn level < 80 μg/dL at baseline as type B (subclinical Zn deficiency), and those with serum Zn level < 60 μg/dL at baseline as type C (Zn deficiency). We investigated the impact of the serum Zn level grading system on the incidence of Com-HEs compared with C–P classification or ALBI grade in a retrospective manner. In addition, factors related to the incidence of Com-HEs were studied in the univariate and multivariate analyses.

This study protocol was in compliance with the 1975 Helsinki Declaration, with approval by the Hyogo college of medicine hospital institutional review board (approval no. 1831).

### 2.4. Statistical Analyses

In the univariate analyses of factors associated with the incidence of Com-HEs, continuous variables were divided into two groups, with the median value as a boundary, and treated as nominal variables. Factors with *p* < 0.05 in the univariate analysis were subjected into the multivariate Cox hazard model. Survival curves were made using the Kaplan–Meier method and compared by the log-rank test. The Akaike information criterion (AIC) with each prognostic model was calculated in order to compare the incidence of Com-HEs. A lower AIC value means better predictability. Data were shown as the median value with an interquartile range (IQR). The significance level in the analysis was set at *p* < 0.05 using the statistical analysis software (JMP ver. 14 (SAS Institute Inc., Tokyo, Japan)).

## 3. Results

### 3.1. Baseline Characteristics

The demographic and clinical features of the analyzed subjects (n = 275) are presented in Table 1. The study cohort included 139 males and 136 females, with a median age (IQR) of 67 years (60.7, 73 years). The median (IQR) follow-up duration was 3.24 years. The median (IQR) serum Zn level was 68.2 μg/dL (57.7, 76.3 μg/dL). There were 52 patients (18.9%) with type A, 148 patients (53.8%) with type B, and 75 patients (27.3%) with type C. With regard to C–P classification and HCV serotype, patients were predominantly C–P A (217/275, 78.9%) and serotype 1 (230/275, 83.6%). There were 124 patients (45.1%) with ALBI grade 1, 142 patients (51.6%) with ALBI grade 2, and 9 patients (3.3%) with ALBI grade 3. During the follow-up period, 198 patients (72.0%) achieved a sustained virological response (SVR). Of these, DAA-based therapies were done in 144 patients and IFN-based therapies were done in 54.

### 3.2. Cumulative Incidence Rate of Com-HEs According to the Serum Zn Level Grading System

**Figure 1:**
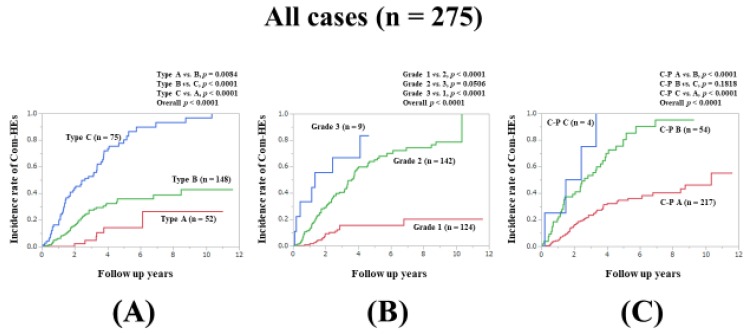
Cumulative incidence rates of composite hepatic events for all cases (n = 275). (**A**) Serum Zn level grading system. (**B**) ALBI grading system. (**C**) Child–Pugh classification.

The incidence of Com-HEs was our primary endpoint. The 3-, 5-, 7-, and 10-year cumulative incidence rate of Com-HEs was 4.83%, 14.21%, 26.47%, and 26.47%, respectively, in patients with type A; 27.48%, 35.76%, 38.68%, and 42.77%, respectively, in patients with type B; and 52.67%, 80.12%, 93.18%, and 96.59%, respectively, in patients with type C (*p* values: *p* = 0.0084 in type A vs. B, *p* < 0.0001 in type B vs. C, and *p* < 0.0001 in type A vs. C; overall *p* value < 0.0001) (Figure 1A).

### 3.3. Breakdown of HEs

The first incidence of any HE was confirmed in 112 patients (40.7%). Of these, HCC development, progression to liver decompensation, appearance or deterioration of ascites, appearance of varices bleeding, appearance of HE, and appearance of ACLF was confirmed in 46, 10, 10, 21, 21, and 4 patients, respectively.

### 3.4. Comparison of Predictability for the Incidence Rate of Com-HEs among the Zn Level Grading System, ALBI Grade, and C–P Classification for All Cases

We compared the predictive accuracy for Com-HEs among three prognostic models for all cases. The cumulative incidence of Com-HEs was well stratified by the serum Zn level grading system, ALBI grade, and C–P classification (overall *p* values, all <0.0001). The prognostic model with the lowest AIC value for Com-HEs among the three models was the Zn level grading system (AIC: 301.788 in Zn level grading system, 303.372 in ALBI grade, and 333.953 in C–P classification) (Figure 1A–C, Table 2).

### 3.5. Subset Analysis 1: Comparison of Predictability for Com-HEs among Three Models in Patients Aged 67 Years or Older (n = 146)

**Figure 2:**
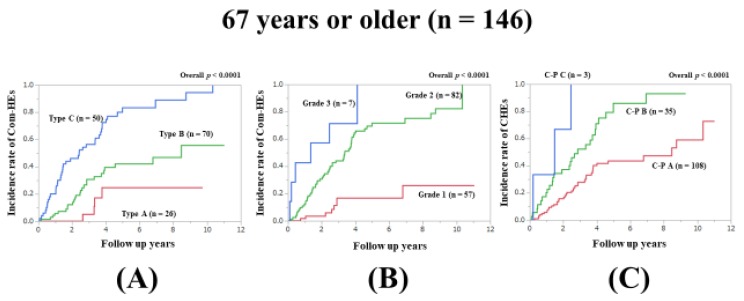
Cumulative incidence rates of composite hepatic events in patients aged 67 years or older (median value in our cohort, n = 146). (**A**) Serum Zn level grading system. (**B**) ALBI grading system. (**C**) Child–Pugh classification.

In patients aged ≥67 years (median value in our cohort), the prognostic model with the lowest AIC value for Com-HEs among the three models was the ALBI grade (AIC: 163.707 in Zn level grading system, 158.999 in ALBI grade, and 187.196 in C–P classification) (Figure 2A–C, Table 2).

### 3.6. Subset Analysis 2: Comparison of Predictability for Com-HEs among Three Models in Patients Aged Less Than 67 Years (n = 129)

**Figure 3:**
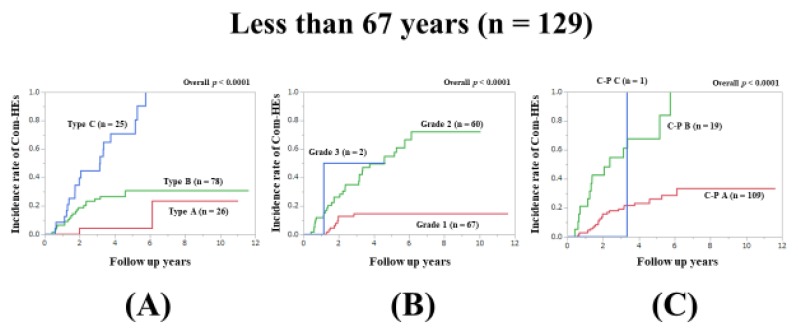
Cumulative incidence rates of composite hepatic events in patients less than 67 years (median value in our cohort, n = 129). (**A**) Serum Zn level grading system. (**B**) ALBI grading system. (**C**) Child–Pugh classification.

In patients aged less than 67 years, the prognostic model with the lowest AIC value for Com-HEs among the three models was the Zn level grading system (AIC: 140.81 in Zn level grading system, 144.943 in ALBI grade, and 147.858 in C–P classification) (Figure 3A–C, Table 2).

### 3.7. Subset Analysis 3: Comparison of Predictability for Com-HEs among Three Models in Patients with FIB4-Index ≥4.46 (n = 138)

**Figure 4:**
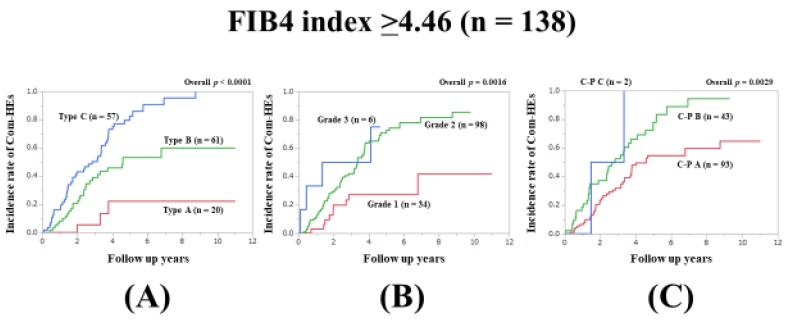
Cumulative incidence rates of composite hepatic events in patients with FIB4 index ≥4.46 (median value in our cohort, n = 138). (**A**) Serum Zn level grading system. (**B**) ALBI grading system. (**C**) Child–Pugh classification.

In patients with FIB4-index ≥4.46 (median value in our cohort), the prognostic model with the lowest AIC value for Com-HEs among the three models was the Zn level grading system (AIC: 156.964 in Zn level grading system, 176.928 in ALBI grade, and 181.478 in C–P classification) (Figure 4A–C, Table 2).

### 3.8. Subset Analysis 4: Comparison of Predictability for Com-HEs among Three Models in Patients with FIB4-Index <4.46 (n = 137)

**Figure 5:**
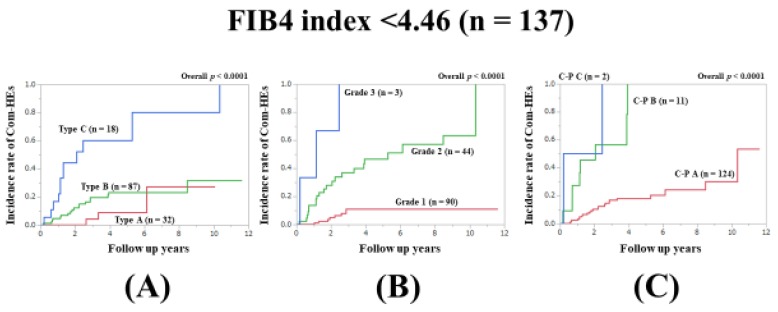
Cumulative incidence rates of composite hepatic events in patients with FIB4 index <4.46 (median value in our cohort, n = 137). (**A**) Serum Zn level grading system. (**B**) ALBI grading system. (**C**) Child–Pugh classification.

In patients with FIB4-index <4.46, the prognostic model with the lowest AIC value for Com-HEs among the three models was the ALBI grade (AIC: 137.715 in Zn level grading system, 121.17 in ALBI grade, and 138.014 in C–P classification) (Figure 5A–C, Table 2).

### 3.9. Subset Analysis 5: Comparison of Predictability for Com-HEs among Three Models in Patients with Body Mass Index (BMI) ≥22.7 kg/m^2^ (n = 139)

**Figure 6:**
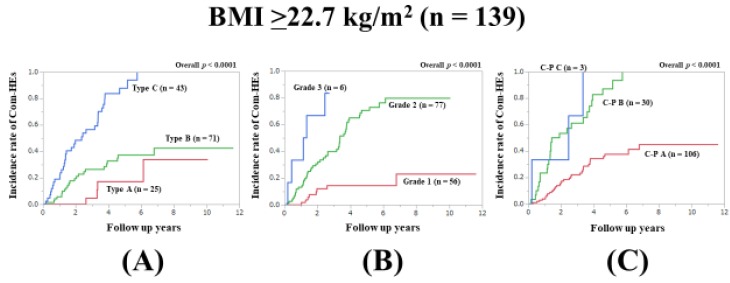
Cumulative incidence rates of composite hepatic events in patients with BMI ≥22.7 kg/m^2^ (median value in our cohort, n = 139). (**A**) Serum Zn level grading system. (**B**) ALBI grading system. (**C**) Child–Pugh classification.

In patients with BMI ≥22.7 kg/m^2^ (median value in our cohort), the prognostic model with the lowest AIC value for Com-HEs among the three models was the Zn level grading system (AIC: 155.801 in Zn level grading system, 157.286 in ALBI grade, and 162.755 in C–P classification) (Figure 6A–C, Table 2).

### 3.10. Subset Analysis 6: Comparison of Predictability for Com-HEs among Three Models in Patients with BMI <22.7 kg/m^2^ (n = 136)

**Figure 7:**
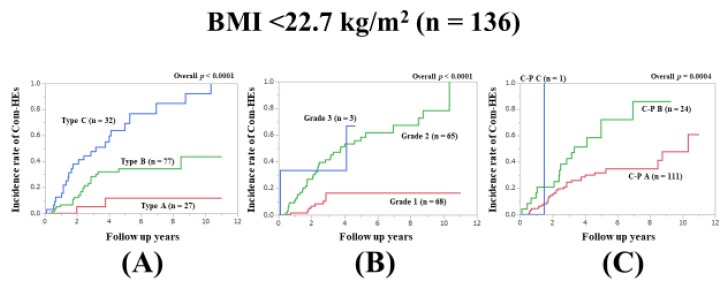
Cumulative incidence rates of composite hepatic events in patients with BMI <22.7 kg/m^2^ (median value in our cohort, n = 136). (**A**) Serum Zn level grading system. (**B**) ALBI grading system. (**C**) Child–Pugh classification.

In patients with BMI <22.7 kg/m^2^, the prognostic model with the lowest AIC value for Com-HEs among the three models was the Zn level grading system (AIC: 150.333 in Zn level grading system, 151.393 in ALBI grade, and 173.036 in C–P classification) (Figure 7A–C, Table 2).

### 3.11. Uni- and Multivariate Analyses of Factors Associated with the Incidence of Com-HEs

The univariate analysis of factors associated with the incidence of CHEs observed 19 factors with *p* < 0.05: Age ≥ 67 years (*p* = 0.0046), male (*p* = 0.0005), C–P classification (*p* < 0.0001), total bilirubin ≥0.9 mg/dL (*p* < 0.0001), serum albumin level <3.9 g/dL (*p* < 0.0001), ALBI grade (*p* < 0.0001), prothrombin time (PT) <76.9% (*p* < 0.0001), platelet count <10.8 × 10^4^/mm^3^ (*p* < 0.0001), aspartate aminotransferase (AST) ≥36 IU/L (*p* < 0.0001), alanine aminotransferase (ALT) ≥28 IU/L (*p* = 0.0041), total cholesterol <151 mg/dL (*p* = 0.0065), triglyceride <78 mg/dL (*p* = 0.0364), serum sodium <140 mmol/L (*p* = 0.0017), serum Zn level grading system (*p* < 0.0001), alpha-fetoprotein ≥5.2 ng/mL (*p* < 0.0001), branched-chain amino acid to tyrosine ratio <4.4 (*p* < 0.0001), absence of SVR (persistent viremia) during the follow-up period (*p* < 0.0001), serum ammonia ≥37 μg/dL (*p* < 0.0001), and FIB-4 index ≥4.46 (*p* < 0.0001). (Table 3) Of these 19 factors, age, AST, ALT, serum albumin, total bilirubin, PT, and platelet count were not included in the multivariate analysis because age, AST, ALT, and platelet count are included in the FIB-4 index; and serum albumin, total bilirubin, and PT are included in the C–P classification. Multivariate analysis for the remaining 12 factors presented that male (*p* = 0.0031), ALBI grade 3 (*p* = 0.0041, ALBI grade 1 as a reference), type B in the serum Zn level grading system (*p* = 0.0238, type A as a reference), type C in the serum Zn level grading system (*p* = 0.0004, type A as a reference), and absence of SVR during the follow-up period (*p* < 0.0001) were significant factors associated with the incidence of Com-HEs. The hazard ratios and 95% confidence intervals of these factors are presented in Table 4.

### 3.12. Serum Zn Level According to HCV Genotype or HCV Viral Load

The difference in serum Zn levels between patients with HCV genotype 1 (n = 230, median (IQR); 69 μg/dL (58, 76.425 μg/dL)) and patients with HCV genotype 2 (n = 37, median (IQR); 65.5 μg/dL (55.65, 73.85 μg/dL)) did not reach significance (*p* = 0.1802). The difference in serum Zn levels between patients with HCV viral load >5 log IU/mL (n = 246, median (IQR); 68.2 μg/dL (58, 75.85 μg/dL)) and patients with HCV viral load <5 log IU/mL (n = 28, median (IQR); 67 μg/dL (52.1, 91.3 μg/dL)) also did not reach significance (*p* = 0.7359).

## 4. Discussion

As mentioned in the introduction section, JSCN defines Zn deficiency, subclinical Zn deficiency, and a normal Zn value based on the serum Zn level [31]. However, the predictability of the serum Zn level grading system in HCV-related LC patients on the incidence of Com-HEs has not been fully investigated, which motivated us to perform the current analysis. In clinical trials, two or more primary endpoints are sometimes adopted, as one endpoint alone may not provide a comprehensive picture of the impacts of the interventions. In such clinical trials, in general, a decision is made as to whether it is ideal to evaluate the synergic effects for all endpoints. The main advantage of using composite outcomes is that a statistically sufficient number of events is ensured [28]. In this study, the first incidence of any HE was identified in 112 patients (40.7%), which seems to be a statistically sufficient event number. Not only HCC but also liver decompensation, ascites, varices bleeding, HE, and ACLF can involve serious consequences in LC patients. Thus, Com-HEs in this study seems to be an appropriate primary outcome measure.

In our previous study, we reported that the serum Zn level grading system proposed by JSCN seems to be helpful for estimating OS in LC patients [31]. In the current results, the serum Zn level grading system proposed by JSCN had the lowest AIC value among the three prognostic models for the incidence of Com-HEs in all cases and in several subgroups. In the multivariate analysis, type B in the serum Zn level grading system and type C in the serum Zn level grading system were revealed as independent factors linked to the incidence of Com-HEs along with male, ALBI grade 3, and persistent viremia. These results imply that the serum Zn level grading system proposed by JSCN can be useful for not only OS but also the incidence of Com-HEs. We believe that the serum Zn level grading system proposed by JSCN has equal or better predictive power than the ALBI grading system. Looking at the Kaplan–Meier curves, type A in the serum Zn level grading system obviously had the best prognosis among type A, B, and C. In LC patients with Zn deficiency, Zn supplementation treatment targeting type A (i.e., 80 μg/dL ≤ serum Zn level < 130 μg/dL) may be beneficial, although an investigation of the impact of Zn supplementation treatment on the incidence of Com-HEs in LC patients was beyond the scope of this study. Further, in all analyses, the C–P classification system had the highest AIC values for the incidence of Com-HEs, which indicates the predictive inferiority for the incidence of Com-HEs compared with the serum Zn level grading system or ALBI grade. These results may be attributed to the inclusion of subjective factors in the C–P classification system.

The advent of DAA agents in patients with HCV allows a virological cure in more than 95% of treated patients, and therefore high rates of decreases of HCV-related complications are also expected in the real-world clinical practice [41,42]. The mechanism for the decreased serum Zn level in HCV-related LC is presumed to involve the non-structural (NS) proteins 3 (NS3, Zn-containing enzyme) and NS5A (Zn metalloprotein) of HCV, but the serum Zn concentration itself may not be affected by the HCV genotype and HCV viral load [43,44,45,46]. Our current results regarding the lack of a relationship between the serum Zn level and HCV genotype or viral load are similar to other reports [45,46]. In the current study, 198 patients (72.0%) achieved SVR during the observation period. Achieving SVR can lead to an increase of serum albumin levels and serum Zn levels [47]. Notwithstanding, the serum Zn level grading system had the lowest AIC among the three assessment methods for all cases and several subgroups, indicating the robustness of the serum Zn level grading system by JSCN on the impact of Com-HEs. In the present study, HCC occurrence or recurrence was identified in 23 patients (16.0%) out of 144 SVR patients treated with DAA-based therapies, whereas it was identified in 6 patients out of 54 SVR patients (11.1%) treated with IFN based-therapies. Although several initial reports with a retrospective nature raised concerns with regard to a possible adverse effect on the risk of HCC occurrence or recurrence after DAA therapy, more recent reports with a prospective nature and systematic reviews have provided evidence that the risk of HCC occurrence or recurrence after DAA therapy is similar or even lower than that observed in patients treated with IFN-based therapies [48]. Further meta-analyses with a bigger cohort will be necessary to assess whether HCV patients with SVR undergoing DAA-based therapies have a lower HCC incidence compared with those undergoing IFN-based therapies.

Several limitations in our study should be mentioned. First, this is a single-center observational study with a retrospective study design, and the impact of the serum Zn level grading system on outcomes should be further tested in another independent study population. Second, the number of our C–P C patients was very small (n = 4) compared with that of C–P A or B patients, introducing bias. Third, only HCV-related LC patients without HCC at baseline were our study cohort; whether the serum Zn level grading system could be extrapolated to HCC patients or non-LC patients or patients with other liver disease etiologies requires further research. Fourth, Zn supplementation treatment in the follow-up period was not taken into consideration in this analysis. Thus, caution should be exercised when interpreting the current study data. However, our study results suggest that the serum Zn level grading system by JSCN is a useful grading system in HCV-related LC patients regarding the incidence of Com-HEs.

In conclusion, the serum Zn level grading system proposed by JSCN seems to be helpful for estimating the incidence of Com-HEs in HCV-related LC patients.

## Figures and Tables

**Table 1:** Baseline characteristics (n = 275).

Variables	All Cases (n = 275)
Age (years)	67 (60.7, 73)
Gender, male/female	139/136
HCV serotype, 1/2/3/ND	230/37/3/5
HCV viral load (log IU/mL), >5/<5/unknown	246/28/1
Child–Pugh classification, A/B/C	217/54/4
Body mass index (kg/m^2^)	22.7 (20.5, 25.3)
Total bilirubin (mg/dL)	0.9 (0.7, 1.3)
Serum albumin (g/dL)	3.9 (3.4, 4.2)
ALBI score	−2.53 (−2.82, −2.06)
ALBI grade, 1/2/3	124/142/9
Prothrombin time (%)	80.4 (69.6, 89.0)
Platelet count (×10^4^/mm^3^)	10.8 (7.2, 15.5)
AST (IU/L)	36 (25, 56)
ALT (IU/L)	28 (18, 47)
Total cholesterol (mg/dL)	151 (134, 179)
Triglyceride (mg/dL)	78 (62, 110)
Fasting blood glucose (mg/dL)	103 (95, 117)
Serum creatinine (mg/dL)	0.66 (0.57, 0.77)
Serum sodium (mmol/L)	140 (139, 141)
Serum Zn level (μg/dL)	68.2 (57.7, 76.3)
Serum Zn level classification, <130, ≥80 μg/dL/<80, ≥60 μg/dL/<60 μg/dL	52/148/75
Branched-chain amino acid to tyrosine ratio	4.4 (3.4, 5.6)
Serum ammonia (µg/dL)	37 (27, 53)
AFP (ng/mL)	5.2 (3.0, 10.1)
FIB-4 index	4.46 (2.54, 7.09)

Data are expressed as median value (interquartile range). HCV; hepatitis C virus, ND; not determined, ALBI; albumin-bilirubin, AST; aspartate aminotransferase, ALT; alanine aminotransferase, Zn; zinc, AFP; alpha-fetoprotein.

**Table 2:** Akaike information criterion value in each prognostic model.

	Serum Zn	ALBI Grade	Child–Pugh
All cases (n = 275)	301.788	303.372	333.953
67 years or older (n = 146)	163.707	158.999	187.196
Less than 67 years (n = 129)	140.81	144.943	147.858
FIB4 index ≥4.46 (n = 138)	156.964	176.928	181.478
FIB4 index <4.46 (n = 137)	137.715	121.17	138.014
BMI ≥22.7 kg/m^2^ (n = 139)	155.801	157.286	162.755
BMI <22.7 kg/m^2^ (n = 136)	150.333	151.393	173.036

Zn; zinc, ALBI; albumin-bilirubin, BMI; body mass index.

**Table 3:** Univariate analyses of factors linked to the incidence of composite hepatic events.

Variables	n	*p* Value
Age ≥67 years, yes/no	146/129	0.0046
Gender, male/female	139/136	0.0005
Child–Pugh classification, A/B/C	217/54/4	<0.0001
Body mass index ≥22.7 kg/m^2^, yes/no	139/136	0.1171
Total bilirubin ≥0.9 mg/dL, yes/no	150/125	<0.0001
Serum albumin ≥3.9 g/dL, yes/no	141/134	<0.0001
ALBI grade, 1/2/3	124/142/9	<0.0001
Prothrombin time ≥76.9%, yes/no	139/136	<0.0001
Platelet count ≥10.8 × 10^4^/mm^3^, yes/no	140/135	<0.0001
AST ≥36 IU/L, yes/no	139/136	<0.0001
ALT ≥28 IU/L, yes/no	142/133	0.0041
Total cholesterol ≥151 mg/dL, yes/no	143/132	0.0065
Triglyceride ≥78 mg/dL, yes/no	141/134	0.0364
Fasting blood glucose ≥103 mg/dL, yes/no	139/136	0.3702
Serum creatinine ≥0.66 mg/dL, yes/no	139/136	0.8082
Serum sodium ≥140 mmol/L, yes/no	174/101	0.0017
Zn, <130, ≥80 μg/dL/<80, ≥60 μg/dL/<60 μg/dL	52/148/75	<0.0001
AFP ≥5.2 ng/mL, yes/no	138/137	<0.0001
BTR ≥4.4, yes/no	135/134	<0.0001
Presence of SVR during the follow-up period, yes/no	198/77	<0.0001
Serum ammonia ≥37 μg/dL, yes/no	147/128	<0.0001
FIB4 index ≥4.46, yes/no	138/137	<0.0001

ALBI; albumin-bilirubin, AST; aspartate aminotransferase, ALT; alanine aminotransferase, Zn; zinc, AFP; alpha-fetoprotein, BTR; branched-chain amino acid to tyrosine ratio, SVR; sustained virological response.

**Table 4:** Multivariate analyses of factors linked to the incidence of Com-HEs.

	HR	95% CI	*p* Value
Gender, male	1.925	1.248–2.970	0.0031
Child–Pugh classification			
Child–Pugh A		Reference	
Child–Pugh B	1.042	0.639–1.699	0.9078
Child–Pugh C	1.078	0.304–3.818	0.8693
ALBI grade			
Grade 1		Reference	
Grade 2	2.499	1.336–4.673	0.0563
Grade 3	2.886	0.972–8.572	0.0041
Total cholesterol ≥151 mg/dL	0.888	0.583–1.352	0.5768
Triglyceride ≥78 mg/dL	0.645	0.415–1.003	0.0514
Serum sodium ≥140 mmol/L, yes/no	0.883	0.587–1.326	0.5476
Serum Zn classification			
Type A (Zn; <130 and ≥80 μg/dL)		Reference	
Type B (Zn; <80, ≥60 μg/dL)	2.715	1.142–6.456	0.0238
Type C (Zn; <60 μg/dL)	4.997	2.044–12.217	0.0004
AFP ≥5.2 ng/mL	1.016	0.612–1.687	0.9506
BTR ≥4.4	0.749	0.431–1.303	0.3066
Presence of SVR	0.428	0.282–0.650	<0.0001
Serum ammonia ≥37 μg/dL	1.223	0.778–1.923	0.3833
FIB4 index ≥4.46	1.134	0.681–1.888	0.6285

HR; hazard ration, CI; confidence interval, ALBI; albumin-bilirubin, Zn; zinc, AFP; alpha-fetoprotein, BTR; branched-chain amino acid to tyrosine ratio, SVR; sustained virological response.

## Abbreviations

LC	liver cirrhosis
HE	hepatic encephalopathy
C-P	Child-Pugh
ALBI	albumin-bilirubin
Com-HEs	composite hepatic events
Zn	zinc
HCC	hepatocellular carcinoma
HCV	hepatitis C virus
JSCN	Japanese society of clinical nutrition
OS	overall survival
AIC	Akaike information criterion
ACLF	acute on chronic liver failure
DAA	direct acting antiviral
IFN	interferon
IQR	interquartile range
SVR	sustained virological response
BMI	body mass index
PT	prothrombin time
AST	aspartate aminotransferase
ALT	alanine aminotransferase
OS	overall survival
NS	non-structural
